# Supplementary material for: Patterns of Intron Gain and Loss in Fungi
Source: PLoS Biol. 2004 Nov 30;2(12):e422. doi: 10.1371/journal.pbio.0020422 (PMC532390; doi:10.1371/journal.pbio.0020422)
Supplement: Table S1 — Also available at http://genes.mit.edu/NielsenEtAl/. (4.3 MB ZIP). [file pbio.0020422.st001.zip › NielsenEtAl/html/417.html]

AN4917.1.NCU00257.1.MG00222.1.FG06588.1


```
 CLUSTAL W (1.82) Multiple Sequence Alignments - Introns Inserted


Sequence 1: MG00222.1	652 aa
Sequence 2: FG06588.1	552 aa
Sequence 3: NCU00257.1	667 aa
Sequence 4: AN4917.1	637 aa
Alignment Length: 868 aa
Number Identitical Residues: 142 aa
Alignment Score (without introns) 9065


MG00222.1 	------------------------------------------------------------
NCU00257.1	MEELLPSAVCPKAAHWTSTFGRYLQEQGYSKSKRVRRVKRSSKSPPASISPATPSDIPPS
FG06588.1 	------------------------------------------------------------
AN4917.1  	------------------------------------------------------------
          	                                                            

MG00222.1 	-------------~----------------------------------------------
NCU00257.1	LPLPPPPPYPRCR1PPPQPNPRDSACMFGNNRLPAFHPGLTQSRIRDTTTGSGLWFPGLR
FG06588.1 	-------------~----------------------------------------------
AN4917.1  	-------------~----------------------------------------------
          	                                                            

MG00222.1 	-------------------------------MAPYTYIQCPCSDPSDLGRSQ---GSSSP
NCU00257.1	SPPLPSESDPANDQLPLPITPEDLYDTPMAPFNPYTYIRCPCSE------------LNPY
FG06588.1 	----------------------------MAPVIPYTYIQCPCSD------------NSPP
AN4917.1  	----------------------------MACPFPYTFISCPCADASVPDPSRKRRSRESP
          	                             :   ***:* ***::.:  . : .  . .. 

MG00222.1 	TQSTPTK-DSNGNLIEEDDEDGRTFDPRAPRSNYSLYPIEYLLYCEDCHQIRCPRCVAEE
NCU00257.1	TKRTPDV-TAQGLSRAAQDDDDHTFDPRAARSNYSLYPLEYLSFCEDCHQIRCPRCVAEE
FG06588.1 	DQQTPQT-PTS-------ASAEHTFDPTDPRSNYSLYPLEYLLYCEDCQQIRCPRCVNEE
AN4917.1  	HKASPVKPRPQPVISDDEDEDEQTFDPRAPRSNFSLYPPEQLLYCEDCHQIKCPRCITEE
          	 : :*  . ..      . .  :****  .***:**** * * :****:**:****: **

MG00222.1 	VVTYFCPSCLFEVPSSNIKSEGNR2CTRSCFQCPICVGILSVTSLE-PSSSSRHLAADNT
NCU00257.1	IVCYYCPNCLFEVPSSNIRSEGSR2CTRSCFQCPICIGPLAVNHVETPPDPNQLLSPDHA
FG06588.1 	VVTYYCPNCLFEVPSSNLRSEGNR2CTRSCYQCPVCIGPTQVMALA-PTE---QQPGADP
AN4917.1  	ILCWYCPSCLFETPSSLVRYEGNR2CGRNCFNCPSCTAPLAVTALQ----------GDPP
          	:: ::**.****.*** :: **.* * *.*::** * .   *  :              .

MG00222.1 	AALPGGPYALNCSYCQWSSLEVGIKFERSNGIYGQLAKLRNGGRPRLSRKEQKERR--EE
NCU00257.1	SSSHSGSYVLSCSYCNWSSTEIGIKFDKPNSIHMQLAKLRNGGETRLTAKERKERR--KE
FG06588.1 	SAHGNSRFALYCQYCNWTSTEIGIDFDRPSGIFTQLSKVNNGGQPRLTAREFKERR--KD
AN4917.1  	SGEQAGPWVLSCNYCMWTTLDIGIKFDKPTNIRAQLSKLTTEPSSTTASRSRGASRSFGD
          	:.   . :.* *.** *:: ::**.*::...*  **:*: .   .  : :.    *:  :

MG00222.1 	SSAFPDSGTGTVPSSIEEEEQKGARESGDAEDDELDVETQFAKLKSFYKSQLADTATSSG
NCU00257.1	QASQGGGGGSS------------TGQGEEDLDTLLDMETQFANLKSFYQNQLSDANGTGK
FG06588.1 	N---PDEPP--------------------PADKDVDAELQYANLKSFYQAQLADTNAS--
AN4917.1  	LKSPLSMYSSIDEQLPSAGTNQPGEEQPTNTHTPRTRDARFAALKAFYKDQMTATNTTGV
          	  :  .   .   .  .   ..   .     .     : ::* **:**: *:: :  :. 

MG00222.1 	SGGLSSLGNLANFGLGSPGSPSMSRLMYMYTGANP-ADTRRKTRMHPMREA~IGPQEGLK
NCU00257.1	VGGDPSAA-LGNLGFDAP--ASLSRIMSLYTGSSSLHDKKSKSRPGTMREA~LAPSEGLQ
FG06588.1 	--GVPSLN--DNLGYGSP--AALTRIMAMYTGHG--HSRKRNGPSDVMREA~LSTEEGLK
AN4917.1  	DPGDFAGQ------FSSP--NTLQRFMVIYT----------QSRLFTLYGT1LTPSEGLF
          	  *  :         .:*   :: *:* :**          :     :  : : ..*** 

MG00222.1 	VLYEPNMKSHEGAIEGGSEETSQNKSKPKNGLDEHAAIVRLRDAG-------WDATTTAE
NCU00257.1	LA----------------------------SLDESSAITALQDFESTTGLDAYSSTASTT
FG06588.1 	VA----------------------------DLDDSAQIKKLHQEG-------LDATATIE
AN4917.1  	IP--------------------------NRPESEVAIIRRLASDDCG-----WDGVASIE
          	:                           .   .: : *  * .  .       ...::  

MG00222.1 	QRASQAPNAAAPVSAQHIRFQDDLRPIPVLLRTKRSKRCPMCRHIMTKPEAKVASNRFRI
NCU00257.1	QLQSQAPYLGASPLHGLTRFTSSLRPIPYLLRTKRSKRCPQCRHIISKPESKVTTTRFRI
FG06588.1 	QNLLQAD---------VQRFQDGLRPIPYLLRTKRSKRCSICRHIISKPENKVTSTRFKI
AN4917.1  	QRTAQPQ-------SFSARFVEDLLPLPTLLRTKRSKRCKSCKHILVKPEFKPQSVRYRI
          	*   *.            ** ..* *:* **********  *:**: *** *  : *::*

MG00222.1 	RLVAGNYIPSISARYLSGGIPPPAEPPGTVAAHHL--LKPLEPVQYLLTLKNPIFESVRV
NCU00257.1	RLIAGNYIPTITIKQL--IIPGLTPPPMPNLPPDT--IEPLKPAQFVLTFKNPIFESVRV
FG06588.1 	KLVAKSYIPTITIRPLNPTAGPVPTTQRPQIQHELP-LKPLTPHHYIITFKNPLFDGIKV
AN4917.1  	KLIAISYIPLPTLRPL--VLPPSASQPLPPPPPDLNSLQPQKPIQLLLTLKNHMFDPVRI
          	:*:* .***  : : *       .    .    .  :::*  * : ::*:** :*: :::

MG00222.1 	SLATPSVTPGRFGSKVTLLCPQFEIGANTD~VWDEVLKDGAPGADASARRRTMVRKPDGE
NCU00257.1	TLATPATTPGRFPAKVTILCPQFEIDSNTD0LKTSPAPQGP-----------KVQAQVVV
FG06588.1 	TLATPNSTPGRFSSKVTILCPQFEIDANTD~MWDDALKDDD---------RDKKRKGEES
AN4917.1  	TLATPSVTPGRVASKVTILCPQFDIGANKD~AWSEALEPVAP----------------PY
          	:****  ****. :***:*****:*.:*.*    .      .                  

MG00222.1 	GGQAEAGKIWEKGRNWVSVVVEIVPASLR-------------------------------
NCU00257.1	NAQALCGRARRGAQVWR-------------------------------------------
FG06588.1 	SGQPEAGKIWERGRNWVSIILEVVPTSLR-------------------------------
AN4917.1  	ERVAEAGKVWEKGRNWTTVVLEVVPGSLPGLGARRSATGTGSNSDDDDDNADNDPDDSED
          	   . .*:  . .: * :   .  . :  . .:  :::.:.:.:......:......:..

MG00222.1 	----VDMLGLGKPASEVDRSPLKEDEDLLEIPMFVRVEWEADAANDDVGQ------AIGK
NCU00257.1	-----------------GRS------------------WDRK------------------
FG06588.1 	----LDDD------KVKDKSPIKEDEDILEIPMFVRMEWEADSQ-QDVGT------ASAK
AN4917.1  	AAKGVSGQFDWSGQASDPSQQIQPDEDVLEIPVFVRMEWDSDSPMESEEGGKDSLGAAAG
          	::.. .     .       .  . ...  . .     .*: .:  ..   ...: .: . 

MG00222.1 	DKDTREKRELAYWCVVGIGRISQD--------
NCU00257.1	--------------------------------
FG06588.1 	EKEGQEKRELAYWCVLGVGRIGSD--------
AN4917.1  	LGVGGTKRELAYWMVLGVGRIVSLSAGSSSSS
          	      . . :     . .   . ::.:::::
```
